# Fatal Case of Tetanus

**Published:** 1869-08

**Authors:** H. K. Steele

**Affiliations:** Dayton, Ohio


### Selections.

---

Fatal Case of Tetanus Resulting from the Removal of Ten Teeth from the Upper Jaw while under the Influence of Nitrous Oxide Gas—By H. K. Steele, M. D., Dayton, Ohio.—John E. P——, aged 19, of strong constitution, robust and in full health, on the 1st of March last, while under the influence of nitrous oxide gas, administered by a Dentist, had ten of the upper teeth removed for the purpose of having a full artificial set inserted.

He felt some of the pain of the operation, but was well able to endure it and recovered apparently from its effects, and continued at his occupation, that of farming. On the 7th of March a twitching of the lower lid of the right eye, with a tendency in it to "draw down," was observed by himself and friends. On the 8th he applied to the Dentist for relief, who made an external application of chloroform, deeming that sufficient. The left eye, however, became similarly affected, and other symptoms were gradually manifested until the 14th, at which time I first saw him (the distance from the city, 7 miles, being probably a reason why I was not sooner called.) There was then inability to separate the jaws more than three-quarters of an inch, a spastic contration of the masseter. There was retraction of the angles of the mouth, and an occasional clonic spasm of the muscles of the abdomen.

Under the influence of a cathartic, with full doses of belladonna and bromide of potassium and ice-bags to the spine; two or three hours sleep was obtained that night, without, however, relaxation of the jaws, or entire subsidence of the abdominal spasm. On the morning of the 15th there was a perceptible exaggeration of the symptoms, the spasms of the abdominal muscles at times being very painful, deglutition performed with some difficulty; a drop of water falling on the chin or running down the neck producing the spasms in their full force.

Chloroform by inhalation moderated the pain and gave temporary comfort. Atropia was substituted for the belladonna and cannabis indica for the potass. bromide with morphine to be given at night.

March 16—Had slept two hours during the night after taking the morphine; but a continuance of it did not maintain relief. This morning the disease is aggravated. He can not remain in bed, and occasionally has to be raised to a standing position, the spasms affecting all the muscles of the body, that of the extensors predominating.

From this time onward the disease increased in severity. The thoracic muscles, those controlling respiration being more affected than the others. There was at no time opisthotonos or emprosthotonos, but the body was powerfully extended to a straight position. He was not able to remain in bed during the last two days, and it was only whilst there was relaxation of the spasms that he could sit in a chair, the rest of the time he was held on his feet and required the windows and doors to be kept open, which, in the very inclement weather, was, of course, an aggravation to his disease and precluded all hopes of affording him relief. He died on the 19th, almost in a standing position, having just sunk down exhausted by the violence of a spasm. The treatment may be summed up as follows:

Ice-bags to spine; morphine; chloroform by inhalation; cannabis indica; potass brom; belladonna; atropia; extract callabar bean by hypodermic injection $\frac{1}{3}$ grain in solution and 1 grain per ore.

The remedies affording the most relief are in the order which they are named.

Chloroform for the last two days affected the respiration dangerously. The hypodermic application of calabar bean was not in the least beneficial.

Dr. John Davis, of Dayton, saw the case with me the last three days.

